# The digit ratio (2D:4D) and economic preferences: no robust associations in a sample of 330 women

**DOI:** 10.1007/s40881-019-00076-y

**Published:** 2019-10-11

**Authors:** Elle Parslow, Eva Ranehill, Niklas Zethraeus, Liselott Blomberg, Bo von Schoultz, Angelica Lindén Hirschberg, Magnus Johannesson, Anna Dreber

**Affiliations:** 1 Department of Economics, Stockholm School of Economics, P.O Box 6501, 11383 Stockholm, Sweden; 2 Department of Economics, University of Gothenburg, Gothenburg, Sweden; 3 Department of Learning, Informatics, Management and Ethics, Karolinska Institutet, Solna, Sweden; 4 Karolinska University Hospital, Karolinska Institutet, Solna, Sweden; 5 Department of Economics, University of Innsbruck, Innsbruck, Austria

**Keywords:** 2D:4D, Economic preferences, Experiment, Testosterone, C91, D03

## Abstract

Many studies report on the association between 2D:4D, a putative marker for prenatal testosterone exposure, and economic preferences. However, most of these studies have limited sample sizes and test multiple hypotheses (without preregistration). In this study we mainly replicate the common specifications found in the literature for the association between the 2D:4D ratio and risk taking, the willingness to compete, and dictator game giving separately. In a sample of 330 women we find no robust associations between any of these economic preferences and 2D:4D. We find no evidence of a statistically significant relation for 16 of the 18 total regressions we run. The two regression specifications which are statistically significant have not previously been reported and the associations are not in the expected direction, and therefore they are unlikely to represent a real effect.

**Electronic supplementary material:**

The online version of this article (10.1007/s40881-019-00076-y) contains supplementary material, which is available to authorized users.

## Introduction

Testosterone has been hypothesised to be associated with a wide range of economic decision making. One aspect of this hypothesis is the theory that prenatal testosterone exposure impacts brain development and therefore can explain some of the heterogeneity in behaviour between individuals. A putative proxy for the level of prenatal testosterone exposure is the ratio of the length of the second digit to the length of the fourth digit (2D:4D) on each hand, as suggested by Manning et al. (1998). Subsequently, many studies have reported associations between 2D:4D and a variety of traits, such as sexual orientation, spatial ability and personality traits, although the results are often conflicting [and with some possibility of publication bias, see, e.g., Puts et al. (2008), Voracek and Loibl (2009), Grimbos et al. (2010), Voracek et al. (2011), but see Hönekopp and Schuster (2010) and Hönekopp and Watson (2011), who do not find evidence for publication bias]. Furthermore, a sizeable literature uses 2D:4D to explore the effect of prenatal testosterone exposure on economic decisions, also with mixed results.

This paper aims to test hypotheses in previous papers in relation to the association between 2D:4D and risk taking, dictator game giving, and the willingness to compete. These preferences are relevant for explaining variation in many economic outcomes. We use a sample of 330 women—which is large given most sample sizes that have previously been reported—in an experiment to measure 2D:4D and economic preferences.

Whilst the 2D:4D measure has been used in many studies, the link between prenatal testosterone and 2D:4D is not strongly established (McIntyre 2006). The oft-cited study by Lutchmaya et al. (2004), which indirectly investigates the link between 2D:4D and prenatal testosterone exposure, finds a statistically significant negative correlation in a sample of 29 children between the testosterone-to-estradiol ratio in amniotic fluid and right hand 2D:4D only, even after controlling for gender (the left hand is reported insignificant). An additional method of investigation is to compare same sex and opposite sex twins, based on the theory of sex-hormone transfer in utero (Miller 1994). van Anders et al. (2006) find that females with a male rather than female co-twin have lower left hand 2D:4D, which the authors argue is due to hormone transfer from male to female foetuses, however, they find no statistically significant results for the right hand. Whilst Voracek and Dressler (2007) in a similar study report a statistically significant result for mean 2D:4D, among studies with much larger sample sizes there is a failure to find statistically significant differences (Hiraishi et al. 2012; Cohen-Bendahan 2005; Medland et al. 2008).^1^ In a study looking at umbilical cord androgen and estrogen concentrations and 2D:4D measured as young adults, Hollier et al. (2015) find no statistically significant association for either hand, using a mixed gender sample of 341 participants. Lastly, other methods of establishing a link between 2D:4D and androgen exposure both post- and peri-natally include using congenital adrenal hyperplasia (CAH) and the CAG repeat polymorphism (McIntyre 2006; Brown et al. 2002), and here also there is a mix of positive and null results.

Even though the link between 2D:4D and prenatal testosterone is not well established, there are many papers investigating the association of 2D:4D with economic decision making. Whilst 2D:4D is an easy-to-measure way to proxy for prenatal testosterone exposure, many of these papers use multiple tests and have relatively small sample sizes. As far as we are aware, none of the previous studies pre-register their analyses. There are often multiple hypotheses involving different ways of measuring the explanatory variable (left hand, right hand, average of both hands or even squared 2D:4D), as well as which controls to include (such as gender, age or sexual orientation) and which subsamples to analyse (such as ethnicity and gender), giving rise to many ‘forking paths’ (Gelman and Loken 2013) and researcher degrees of freedom (Simmons et al. 2011). As discussed in Simmons et al. (2011), researchers have many options available in choosing among outcome variables, controls and subsample selection, creating ambiguity in the research process and potentially generating higher rates of false positives than 5%, even if researchers do not intend to do so. In our review of the literature in the following subsections, we consider statistically significant results to be cases where the *p* value is less than 0.05 and report anything above that threshold as insignificant, as is typically used. We present tables to summarise the results of studies that use comparable measures of economic preferences to our experiments.^2^ However, in our own results in this paper, we instead consider a *p* value less than 0.05 to indicate suggestive evidence, whilst statistical significance requires a *p* value less than 0.005, following Benjamin et al. (2018).

Benjamin et al. (2018) suggest a change in the *p* value defining statistically significant new discoveries from 0.05 to 0.005, to improve the reproducibility of scientific studies (in terms of reducing rates of false positives). The authors propose that where *p* values are below 0.05 but above 0.005, this should be interpreted as suggestive evidence. Whilst our study aims to be a replication of past studies, the results of past studies are mixed and therefore we think it is appropriate to use the more conservative 0.005 threshold for statistical significance. An additional motivation for a more conservative threshold than 0.05 is that we, following the existing literature, run several tests for each outcome measure.

### Dictator game giving

Several papers have looked at the relationship between 2D:4D and giving in the dictator game.^3^ The dictator game removes any repercussions of failure to reciprocate (unlike the ultimatum game), and in all the below studies the participants were told that the recipient in the game is another participant whose identity is unknown.^4^ The hypothesised relationship between 2D:4D and dictator game giving is positive, with higher exposure to testosterone (low 2D:4D) being associated with lower levels of dictator game giving. The results from studies using the dictator game are summarised in Table 1, showing that insignificant findings are common. When statistically significant, regressions using squared 2D:4D measures find an inverse U-shaped relationship between 2D:4D and dictator game giving (low dictator game giving is associated with both low and high testosterone). From the five previous papers summarised in Table 1, 2 out of the 43 total tests find statistically significant positive results, 1 out of 43 finds statistically significant negative results, 8 out of 43 find an inverse U-shaped relationship and 32 out of 43 find no statistically significant results (where here significance is $p<0.05$).

**Table 1:** Dictator game giving studies

Study	Men						Women						Both sexes					
	L	L	R	R	M	M	L	L	R	R	M	M	L	L	R	R	M	M
		$\hbox {L}^2$		$\hbox {R}^2$		$\hbox {M}^2$		$\hbox {L}^2$		$\hbox {R}^2$		$\hbox {M}^2$		$\hbox {L}^2$		$\hbox {R}^2$		$\hbox {M}^2$
Millet and Dewitte (2009), Study 1 neutral prime															S− (119$\hat{)}$			
Millet and Dewitte (2009), Study 1 aggressive prime															NS (119$\hat{)}$			
Millet and Dewitte (2009), Study 2 neutral prime															NS (90$\hat{)}$			
Millet and Dewitte (2009), Study 2 aggressive prime															S+ (90$\hat{)}$			
Buser (2012)													NS (221)		NS** (221)		NS** (221)	
Brañas-Garza et al. (2013), 2010 study		S+, S− (87)		S+, S− (88)				NS, NS (61)		S+, S− (61)			NS* (170)	S+, S− (170)	NS* (171)	S+, S− (171)		
Brañas-Garza et al. (2013), 2011 study		NS, NS (68)		NS, NS (69)				NS, NS (53)		S+, S− (53)			NS* (126)	NS, NS (126)	S+* (127)	S+, S− (127)		
Galizzi and Nieboer (2015), all													NS (602)	NS, NS (602)	NS (602)	NS, NS (602)		
Galizzi and Nieboer (2015), Caucasian													NS (201)	NS, NS (201)	NS (201)	S+, S− (201)		
Galizzi and Nieboer (2015), Chinese													NS (221)	NS, NS (221)	NS (221)	NS, NS (221)		
Galizzi and Nieboer (2015), South Asian													NS (81)	NS, NS (81)	NS (81)	NS, NS (81)		
Brañas-Garza et al. (2019)													NS (560)	NS, NS (560)	NS (560)	NS, NS (560)		

Sample sizes in parentheses

*L* left hand, *R* right hand, *M* mean of left and right hands, *NS* not statistically significant, *S+* statistically significant positive relationship, *S*− statistically significant negative relationship

*Does not control for gender, **Statistically significant positive result for binary variable where 1 indicates 4D longer and 0 indicates all other scenarios (i.e., same as 2D

or shorter), authors note results do not change for genders separately, they also control for age, nationality and experience in previous games

${}^{\hat{\,}}$Sample size is the total sample for that study, the authors do not state the sample split for neutral or aggressive prime groups

### Risk taking

While several review papers find that women are on average more risk averse than men, [see, e.g., Eckel and Grossman (2008), Croson and Gneezy (2009), Charness and Gneezy (2012)], there is also evidence from a meta-analysis by Nelson (2015) suggesting that the difference (in terms of effect size) is not very large. Nevertheless, there is a substantial literature looking into a biological explanation for this gender difference through prenatal testosterone exposure and the 2D:4D ratio. As far as we are aware, only one study finds an association between 2D:4D and risk tasking in men and not in women (Stenstrom et al. 2011). The hypothesis is that risk taking is negatively related to 2D:4D—higher testosterone exposure is associated with higher risk taking (and lower risk aversion). The results from studies using risk taking tasks are summarised in Table 2. We limit our analysis of the previous literature to the areas of financial or general risk taking. There are numerous ways to measure risk-taking in experimental tasks, as well as the digit ratio (such as by scanner, or calliper etc.), which can add measurement error. From the 18 previous papers summarised in Table 2, 1 out of the 109 total tests finds positive statistically significant results, 15 out of 109 find negative statistically significant results, and 93 out of 109 find no statistically significant results (significance here is $p<0.05$).

**Table 2:** Risk-taking studies

Study	Men					Women					Both sexes				
	L	L	R	R	M	L	L	R	R	M	L	L	R	R	M
		$\hbox {L}^2$		$\hbox {R}^2$			$\hbox {L}^2$		$\hbox {R}^2$			$\hbox {L}^2$		$\hbox {R}^2$	
Dreber and Hoffman (2007), study 1											S− (120)		NS (116)		
Dreber and Hoffman (2007), study 2											NS (116)		NS (115)		
Apicella et al. (2008)	NS (85)		NS (88)												
Sapienza et al. (2009)	NS (116)		NS (116)		NS (116)	NS (65)		NS (65)		NS (65)	NS (181)		NS (181)		NS (181)
Coates and Page (2009)			S− (47)												
Brañas-Garza and Rustichini (2011), risk aversion			NS (72)					S+ (116)					NS (188)		
Brañas-Garza and Rustichini (2011), combined risk aversion			S− (72)					NS (116)					NS (188)		
Garbarino et al. (2011)*															S− (151)
Stenstrom et al. (2011), financial risk${}^{\hat{\,}}$			S− (219)					NS (194)							
Sytsma (2014), gain domain*	NS (105)		NS (98)		NS (92)	S− (29)		S− (24)		NS (23)	S− (134)		NS (122)		NS (115)
Sytsma (2014), loss domain*	S− (105)		NS (98)		NS (92)	NS (29)		NS (24)		NS (23)	NS (134)		NS (122)		NS (115)
Sytsma (2014), average*	S− (105)		NS (98)		NS (92)	NS (29)		NS (24)		NS (23)	S− (134)		NS (122)		NS (115)
Aycinena et al. (2014)	NS (106)	NS, NS (106)	NS (106)	NS, NS (106)		NS (78)	NS, NS, (78)	NS (78)	NS, NS, (78)		NS (184)	NS, NS (184)	NS (184)	NS, NS (184)	
Drichoutis and Nayga (2015)														NS, NS (138)	
Schipper (2014), gains*			NS (103)					NS (71)							
Schipper (2014), losses*			NS (111)					NS (80)							
Schipper (2014), gains white*			NS (47)					NS (25)							
Schipper (2014), losses white*			NS (50)					NS (27)							
Schipper (2014), gains asian*			NS (52)					NS (41)							
Schipper (2014), losses asian*			NS (56)					NS (48)							
Bönte et al. (2016), investment risk${}^{\hat{\,}}$ *													NS (432)		
Bönte et al. (2016), general risk${}^{\hat{\,}}$ *													S− (432)		
Barel (2017), general risk${}^{\hat{\,}}$											NS (211)		S− (211)		
Barel (2017), financial risk${}^{\hat{\,}}$											NS (211)		NS (211)		
Chicaiza-Becerra and Garcia-Molina (2017), full											NS (123)		NS (123)		
Chicaiza-Becerra and Garcia-Molina (2017), midland											NS (115)		NS (115)		
Brañas-Garza et al. (2018), risk preference											S− (664)		S− (664)		
Brañas-Garza et al. (2018), general risk attitude${}^{\hat{\,}}$											NS (704)		NS (704)		
Lima de Miranda et al. (2018)											NS (144)	NS, NS (144)	NS (145)	NS, NS (145)	
Alonso et al. (2018)											NS (390)		NS (390)		
Neyse et al. (2019), gains Germany*$^+$											NS (181)		NS (183)		
Neyse et al. (2019), losses Germany*$^+$											NS (185)		NS (187)		
Neyse et al. (2019), mixed Germany*$^+$											NS (188)		NS (188)		
Neyse et al. (2019), gains Vietnam*$^+$											NS (162)		NS (162)		
Neyse et al. (2019), losses Vietnam*$^+$											NS (162)		NS (161)		
Neyse et al. (2019), mixed Vietnam*$^+$											NS (162)		NS (162)		

Sample sizes in parentheses

*L* left hand, *R* right hand, *M* mean of left and right hands, *NS* not statistically significant, *S+* statistically significant positive relationship, *S*− statistically significant negative relationship

*Multiple other controls, ${}^{\hat{\,}}$Questionnaire elicitation of risk, $^+$Sample size refers to number of subjects

### Competitiveness

Whilst there is evidence for gender differences in self-selection into competition (Niederle and Vesterlund 2007; Dariel et al. 2017),^5^ there exists substantially less literature looking at the relation between prenatal testosterone exposure and willingness to compete, relative to the other economic preferences discussed. Given the gender differences observed in this scenario, the hypothesis tested in the existing literature is that higher testosterone is associated with higher competitiveness, leading to a negative relationship between 2D:4D and the willingness to compete. Table 3 summarises the results from previous studies. Out of the 10 total tests reported across previous studies, 2 find statistically significant negative results and 8 find no statistically significant results (here significance is $p<0.05$).

**Table 3:** Competitiveness studies

Study	Men						Women						Both sexes					
	L	L	R	R	M	M	L	L	R	R	M	M	L	L	R	R	M	M
		$\hbox {L}^2$		$\hbox {R}^2$		$\hbox {M}^2$		$\hbox {L}^2$		$\hbox {R}^2$		$\hbox {M}^2$		$\hbox {L}^2$		$\hbox {R}^2$		$\hbox {M}^2$
Apicella et al. (2011)*	NS (83)		NS (86)															
Bönte et al. (2017), study 1 behavioural measure													NS (461)		NS (461)			
Bönte et al. (2017), study 1 self-reported measure													NS (461)		S− (461)			
Bönte et al. (2017), study 2 behavioural measure													NS (150)		NS (150)			
Bönte et al. (2017), study 2 self-reported measure													NS (618)		S− (618)			

Sample sizes in parentheses

*L* left hand, *R* right hand, *M* mean of left and right hands, *NS* not statistically significant, *S+* statistically significant positive relationship, *S*− statistically significant negative relationship

*Study includes a control for sexual orientation

## Method

### Experimental procedures and design

The data on 2D:4D were collected in conjunction with a study on the influence of the oral contraceptive pill (Ranehill et al. 2017). The pre-analysis plan specifying the analysis prior to completion of data collection for this study was posted on the Open Science Framework website on the 21st of August 2015 (available at http://osf.io/he8nb/). However, the 2D:4D measure was not part of the main planned analyses in this double-blind randomised study. The exact analyses for the 2D:4D measure were therefore not specified in the pre-analysis plan. Instead it was stated in the pre-analysis plan that the 2D:4D data would be used to carry out tests of previous 2D:4D results reported as statistically significant in the literature (i.e., the data were collected to be able to replicate previous findings). The previously reported results in the literature are therefore the starting point for our analyses, but ideally our tests should have been exactly specified in the pre-analysis plan.

The participants in the study were 340 healthy women aged 18–35 years recruited following the criteria used in the oral contraceptive study.^6^ Participants in this study thus had agreed to participate in a randomized controlled trial on the effects of the contraceptive pill. Participants participated in two sessions for the overall study: once at baseline, and once during the follow-up (the end of the study medication treatment period). Both sessions took place at the Karolinska University Hospital. The economic experiment was performed during the second session. During both sessions, we first collected blood samples for the participants before they filled out surveys on sexual function, general well-being and depressive symptoms. Participants then filled out a survey on facial preferences. In the second session, participants participated in the economic experiment after the survey of facial preferences. The economic experiment was computerized.^7^ The economic part took about 30 minutes, while the other parts took about 20 minutes. Participants were not informed about their earnings for any task during the experiment but were paid at a later date (within 2 months after having participated in the experiment).

For details on how participants were recruited, the criteria for inclusion and exclusion, and further sample characteristics see Ranehill et al. (2017). Approximately 60% of participants reported an education level of university studies (ongoing) or a university degree. Unfortunately, we do not have ethnicity data for our sample of participants. While the majority of the participants were Caucasian, we cannot rule out that controlling for ethnicity would affect our results. The statistical analysis is based on 330 participants as 10 participants did not complete the data collection (7 discontinued treatment and thus did not complete the data collection, and 3 had missing hand measurements).

The economic experiments on decision making were also reported and analysed in Ranehill et al. (2017). The tests measured dictator game giving, financial risk taking, and willingness to compete. The order of the experimental tasks was kept constant across all participants, starting with the dictator game, the risk task, and thereafter the three stages of the competitiveness task.^8^ Participants were not informed about their earnings for any task during the experiment but were paid at a later date (within 2 months after having participated in the experiment). The economic experiment was computerized and took about 30 minutes.

The dictator game giving measure was elicited in a modified dictator game where the participant was asked to allocate SEK 100^9^ between herself and a charitable organization, repeated five times with a different charity organisation in each repetition. The average donation across the five decisions is used as our measure of dictator game giving. We include five dictator game decisions to reduce measurement error.

We measure risk taking with repeated lottery choices, involving 18 decisions between a certain payoff, and a 50:50 gamble to win either a larger amount of money than the safe option or SEK 0. The certain payoff amounts varied from SEK 40 to 280, and the gamble amounts were either SEK 200, 300 or 400. The percentage of choices of the gamble (i.e., the number of times the gamble was chosen over the certain payoff) is used as our measure of risk taking.

Measuring willingness to compete consisted of asking participants to solve simple tasks of adding numbers for 3 minutes, first under a non-competitive piece-rate payment scheme of SEK 5 for each correct answer, and then under a competitive tournament payment scheme of SEK 10 for each correct answer only if more tasks were solved than a random competitor (a participant selected from a previous session), otherwise the pay was zero (with SEK 5 for each person in the case of a tie). Then, in the last part, the participant could select to be paid either under the non-competitive piece rate scheme or the competitive tournament scheme. For our willingness to compete measure, we used the choice of competitive tournament scheme in this part (dummy variable where 1 is choice of competitive tournament scheme).

2D:4D results in the literature are sometimes presented for the left hand, sometimes for the right hand, and sometimes for the average of both hands. Following the existing literature, we therefore present results for all these three 2D:4D measures. In the literature results are sometimes presented for a linear model and sometimes a squared term is added to allow for a non-linear relationship. Following the existing literature, we therefore present results both without (the linear model) and with a quadratic term. In total we therefore estimate 18 regression models; 6 models for each outcome measure. In the models with a squared term we evaluate the significance of 2D:4D as the significance of the regression coefficient for the squared 2D:4D, but we also report the significance of an F-test for the joint significance of 2D:4D and the squared 2D:4D.

### Power calculations

We first estimate our power to detect previous statistically significant results, based on all statistically significant findings in the literature (for models without the squared term and where the necessary information was available) and we have the following ranges of power calculations. For dictator game giving, the range of power is 0.896 to 0.999 with a mean of 0.941 at the 5% level and 0.656 to 0.994 with a mean of 0.791 at the 0.5% level. For risk taking, the range of power is 0.423 to 0.999 with a mean of 0.748 at the 5% level and 0.148 to 0.999 with a mean of 0.535 at the 0.5% level. For the willingness to compete, the range is 0.441 to 0.468 with a mean of 0.454 at the 5% level and 0.159 to 0.176 with a mean of 0.167 at the 0.5% level. However, we note that there are drawbacks to doing such power calculations, since it is very likely that original results are biased in terms of being exaggerated even if they are true positives [see, e.g., Gelman and Carlin (2014)]. Lastly, with our sample size of 330, we have 90% power to find a small effect size of $r = 0.17$ with $\alpha = 0.05$, and $r = 0.22$ with $\alpha = 0.005$.

### Measuring 2D:4D

Digit measurement expressed in millimetres (mm) was performed for digit two (2D) and digit four (4D), using a Vernier digital calliper 0–150 mm (USA, Cocraft) with a precision of 0.01mm. Digit length was directly measured by two raters from the mid-point of the proximal crease of the proximal phalanx to the distal tip of the distal phalanx for 2D and 4D on both left and right hand. The reliability of direct measurement of digits was tested, demonstrating a high repeatability and differences between subjects greater than measurement errors (Savic et al. 2017). The mean value of two measurements of the 2D and 4D length was calculated and then divided to create the 2D:4D ratio, which was used for further statistical analysis.

## Results

Overall we report results for 18 regression variations, with 6 different specifications for the explanatory variables run separately using OLS for the 3 dependent variables, representing our outcome measures of dictator game giving, risk taking, and the willingness to compete. We note that the Pearson correlation between left and right hand 2D:4D in our sample is 0.63.^10^ Table 4 shows the means and standard deviations for the 2D:4D measures and the outcome variables.

**Table 4:** Summary statistics

	Mean	SD
Giving	40.748	30.356
Risk	0.550	0.186
Comp.	0.424	0.495
2D:4D LH	0.967	0.033
2D:4D RH	0.980	0.031
2D:4D Avg	0.973	0.029
2D:4D LH sqr	0.935	0.063
2D:4D RH sqr	0.961	0.062
2D:4D Avg sqr	0.948	0.056
Observations	330	

We report the regression results in the following three tables, grouped by outcome measure. Table 5 shows the results for the dictator game giving measure, whilst Table 6 shows risk taking and Table 7 shows the willingness to compete as the dependent variable.

**Table 5:** Dictator game giving results

	(1)	(2)	(3)	(4)	(5)	(6)
	Giving	Giving	Giving	Giving	Giving	Giving
2D:4D LH	23.2			− 930.5		
	(50.87)			(1655.11)		
2D:4D RH		6.50			3729.8	
		(55.70)			(1993.72)	
2D:4D Avg			18.7			2648.6
			(57.81)			(2537.98)
2D:4D LH sqr				491.8		
				(854.80)		
2D:4D RH sqr					− 1892.6	
					(1012.69)	
2D:4D Avg sqr						− 1350.6
						(1306.45)
Constant	18.3	34.4	22.5	480.1	− 1795.0	− 1256.6
	(49.20)	(54.62)	(56.29)	(800.98)	(981.03)	(1232.14)
N	330	330	330	330	330	330
F	0.21	0.014	0.11	0.25	1.75	0.63
p	0.65	0.91	0.75	0.78	0.18	0.53

This table reports OLS regressions for six specifications where the dependent variable is a measure for dictator game giving (the average donation across the five decisions). LH, RH and Avg correspond to left hand, right hand and average of both hands 2D:4D, respectively. LH sqr, RH sqr and Avg sqr correspond to the square of the left, right and average of both hands 2D:4D measures, respectively. The lower panel shows the *F* statistic and the *p* value from a test of the significance of each regression model, and the sample size *N*. Robust standard errors in parentheses.

*$$p<0.05$$, **$$p<0.005$$

**Table 6:** Risk-taking results

	(1)	(2)	(3)	(4)	(5)	(6)
	Risk	Risk	Risk	Risk	Risk	Risk
2D:4D LH	0.42			− 6.21		
	(0.31)			(11.15)		
2D:4D RH		0.29			27.3	
		(0.34)			(13.89)	
2D:4D Avg			0.44			16.0
			(0.36)			(16.01)
2D:4D LH sqr				3.42		
				(5.75)		
2D:4D RH sqr					− 13.7	
					(7.04)	
2D:4D Avg sqr						− 7.99
						(8.22)
Constant	0.14	0.27	0.12	3.35	− 13.0	− 7.45
	(0.30)	(0.34)	(0.35)	(5.41)	(6.85)	(7.79)
N	330	330	330	330	330	330
F	1.83	0.69	1.48	1.13	2.13	1.23
p	0.18	0.41	0.23	0.32	0.12	0.29

This table reports OLS regressions for six specifications where the dependent variable is a measure for risk taking (the percentage of choices of the gamble). LH, RH and Avg correspond to left hand, right hand and average of both hands 2D:4D, respectively. LH sqr, RH sqr and Avg sqr correspond to the square of the left, right and average of both hands 2D:4D measures, respectively. The lower panel shows the F statistic and the p-value from a test of the significance of each regression model, and the sample size N. Robust standard errors in parentheses.

* $$p<0.05$$, ** $$p<0.005$$

**Table 7:** Willingness to compete results

	(1)	(2)	(3)	(4)	(5)	(6)
	Comp.	Comp.	Comp.	Comp.	Comp.	Comp.
2D:4D LH	1.19			73.4**		
	(0.80)			(22.83)		
2D:4D RH		0.76			43.2	
		(0.86)			(36.33)	
2D:4D Avg			1.21			97.3**
			(0.92)			(33.93)
2D:4D LH sqr				− 37.2**		
				(11.84)		
2D:4D RH sqr					− 21.6	
					(18.46)	
2D:4D Avg sqr						− 49.3**
						(17.44)
Constant	− 0.73	− 0.32	− 0.76	− 35.7**	− 21.2	− 47.5**
	(0.78)	(0.85)	(0.89)	(11.00)	(17.87)	(16.49)
N	330	330	330	330	330	330
F	2.21	0.77	1.74	7.46	1.07	5.22
p	0.14	0.38	0.19	0.00068	0.34	0.0059

Notes: This table reports OLS regressions for six specifications where the dependent variable is a binary measure for the willingness to compete (the value 1 represents choosing the competitive tournament scheme). LH, RH and Avg correspond to left hand, right hand and average of both hands 2D:4D, respectively. LH sqr, RH sqr and Avg sqr correspond to the square of the left, right and average of both hands 2D:4D measures, respectively. The lower panel shows the F statistic and the p-value from a test of the significance of each regression model, and the sample size N. Robust standard errors in parentheses.

*$$p<0.05$$, **$$p<0.005$$

**Fig. 1:**
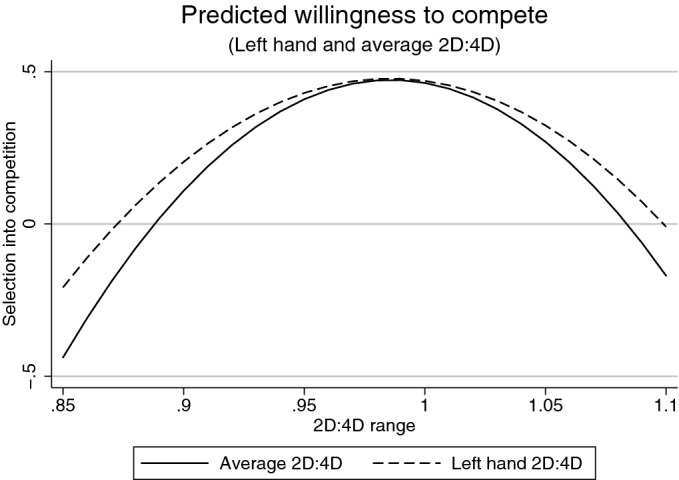
Plot of the predicted relationships between 2D:4D and willingness to compete, for the regression with left hand 2D:4D and left hand 2D:4D squared, and also for the regression with average 2D:4D and average 2D:4D squared

We find no evidence of a statistically significant relation between 2D:4D and either dictator game giving or risk taking ($p>0.05$). For competitiveness we find no evidence in the linear models either ($p>0.05$). When we add a squared term we find statistically significant evidence ($p<0.005$ for the squared 2D:4D coefficient) in both the regression for left hand 2D:4D and competitiveness, and the regression for the average 2D:4D of the two hands and competitiveness.^11^ However, these regression specifications are not among those that have previously been reported in the literature for the willingness to compete. We plot the predicted relationships from these statistically significant specifications to illustrate the interpretation of the predicted relationships, using the range of 2D:4D that we see in our data (Fig. 1).^12^

The willingness to compete outcomes predicted by our regression equations show an inverse U-shaped relationship where, across a range of 2D:4D values from 0.85 to 1.1, low 2D:4D (synonymous with high prenatal testosterone exposure) predicts low competitiveness, which does not fit with the pre-existing hypothesis that high testosterone correlates with high competitiveness.^13^ The highest willingness to compete is instead associated with mid-range 2D:4D for this predicted relationship. If the hypothesis tested in the existing literature was to hold here, we would see a decreasing relationship. As most 2D:4D measurements are below 1, we see that most of the distribution of observations would lie to the left of the peak, in the region of an increasing relationship, which is the opposite to the hypothesised relationship. The estimated inverse U-shaped relationship is thus unlikely to represent a real effect.

## Discussion

In this study we find little evidence of 2D:4D correlating with economic preferences in a sample of 330 women. The only two statistically significant regression specifications ($p<0.005$) are not in the hypothesised direction and are not consistent with any previous findings, and are thus likely to be a false positive. The study by Ranehill et al. (2017) that was run in conjunction, but looking at the effect of the oral contraceptive pill, also did not find any impact of the pill on economic preferences.

Our null results could be due to several reasons. First, 2D:4D may be a reliable proxy of prenatal testosterone exposure but prenatal testosterone exposure may not correlate with economic preferences and previous results are false positive results. Second, 2D:4D may be a reliable proxy of prenatal testosterone but the relation between prenatal testosterone exposure and economic preferences is so weak that with 330 women we do not have sufficient statistical power to detect true positive results. Third, 2D:4D may be a weak or noisy proxy of prenatal testosterone but the relation between prenatal testosterone exposure and economic preferences is actually strong; but again we could then be underpowered to detect true positive results. Fourth, 2D:4D may be a weak or noisy proxy of prenatal testosterone and there is also a weak relation between prenatal testosterone exposure and economic preferences; again we could then be underpowered to detect true positive results. Fifth, 2D:4D may not correlate with economic preferences among women, thus our study would be set up to not find anything since we have only women in our sample. Given previous literature it is not clear to us why this should make a difference but additional high-powered studies, with pre-analysis plans, on men or mixed gender would be useful.

Sixth, perhaps there is something special about our sample that makes us not find a true correlation between 2D:4D and economic preferences that exist in more general samples. The editor pointed out that the selection of women who are non-smokers and who are willing to use oral contraceptives might generate a sample that is more risk-averse than the general population, or have a higher 2D:4D ratio. With respect to risk taking, the closest comparison of our sample to the general population is Boschini et al. (2018) who explore risk preferences in a random sample of 487 Swedish women in a similar risk preference elicitation task of choices over lotteries versus safe options. In these samples, the average switching point is very similar—just below the risk neutral point. With respect to 2D:4D, our sample is within a similar range to previous studies.^14^ In sum, more work is needed to disentangle these six possible explanations for our null results.

In a related vein, the evidence linking sex hormone administration to economic preferences is also inconclusive with most studies failing to reject the null hypothesis of no effect. The few statistically significant findings (as well as the null results) need, however, to be interpreted with caution because of low statistical power and the many researcher degrees of freedom [see the recent review by Dreber and Johannesson (2018) for more information].

In sum, more work is needed with larger sample sizes and pre-registered hypotheses to have enough statistical power to find small effects of 2D:4D on economic preferences. Additionally, studies using improved indicators of prenatal testosterone exposure may be warranted.

## Electronic supplementary material

Below is the link to the electronic supplementary material.

Supplementary material 1 (pdf 211 KB)

Supplementary material 2 (pdf 313 KB)

## Footnotes

1 Studies finding statistically significant results have sample sizes of 24 and 28 (van Anders et al. 2006 and Voracek and Dressler 2007 respectively) whereas studies finding no statistically significant differences report sample sizes of 55, 55 and 449 (Cohen-Bendahan 2005; Hiraishi et al. 2012 and Medland et al. 2008, respectively).

2 The results we include in the table for mixed gender samples are specifications which include a gender control only, unless noted otherwise.

3 Some studies have looked at other games, such as public goods games, and interpret behaviour as altruistic or pro-social, such as Millet and Dewitte (2006).

4 However, Millet and Dewitte (2009) use a hypothetical dictator game.

5 Although there is some evidence that this effect is context dependent (Gneezy et al. 2009)

6 Such as having a body mass index between 19–30, willing to start using oral contraceptives, being fluent in the Swedish language, being a non-smoker, not being pregnant and so on.

7 See online appendix for the economic experiment instructions as well as the instructions for the non-economic parts.

8 The order is constant for all participants for logistical reasons—the experiment was performed at the university hospital by the hospital staff over a period of several years, and randomizing the order of tasks was not something the staff wanted.

9 SEK 100 corresponds to roughly USD 11.

10 Whilst similar correlations have been found previously [see, e.g., Brañas-Garza et al. (2019)], our correlation appears to be on the low side (e.g., Neyse et al. (2016) find correlations of 0.727 for men and 0.765 for women), however, other studies also find even lower correlations (e.g., Bönte et al. (2017)).

11 We also perform two additional analyses for the regression with willingness to compete and 2D:4D, which were not pre-specified in our pre-analysis plan. We include these in the online appendix Tables 8 and 9. The first analysis includes risk taking as a control variable. We find that adding this control variable leads to no change in the qualitative conclusions, except for the regression of average 2D:4D with the squared term, which now shows only suggestive evidence of a relation. The second analysis adds the piece-rate task performance as a control variable. In this case the regressions with left hand and left hand squared, and average squared now indicate suggestive evidence of a relation, as the *p* values for the coefficients are $p<0.05$. The results for the other regressions do not change.

12 Our range of 2D:4D is within that commonly seen in the literature of around 0.8–1.2.

13 We note that some of the predicted values for the willingness to compete are negative, which is an implication of using OLS for estimation.

14 Neyse et al. (2016) find that women in their sample on average have a left hand 2D:4D of 0.970 (with standard deviation of 0.0341) and for right hand 2D:4D the average is 0.967 (with standard deviation of 0.0362). Brañas-Garza et al. (2018) find that in their female subsample the average left hand 2D:4D 0.9733 (with standard deviation of 0.0321) while average right hand 2D:4D is 0.9770 (with standard deviation of 0.0325). Our results are average left hand 2D:4D of 0.9667 (with standard deviation of 0.0327) and average right hand 2D:4D of 0.9801 (with standard deviation of 0.0313).
