# Supplementary material for: The digit ratio (2D:4D) and economic preferences: no robust associations in a sample of 330 women
Source: J Econ Sci Assoc. 2019 Oct 11;5(2):149–69. doi: 10.1007/s40881-019-00076-y (PMC6917633; doi:10.1007/s40881-019-00076-y)
Supplement: Supplementary file 1 — Supplementary material 1 (pdf 211 KB) [file 40881_2019_76_MOESM1_ESM.pdf]

A Online Appendix

A.1 Additional analyses

Table 8: Willingness to compete results, controlling for risk taking

	(1) Comp.	(2) Comp.	(3) Comp.	(4) Comp.	(5) Comp.	(6) Comp.
2D:4D LH	0.91			77.6**		
	(0.80)			(22.32)		
Risk	0.67**	0.68**	0.67**	0.68**	0.67**	0.66**
	(0.13)	(0.13)	(0.13)	(0.12)	(0.13)	(0.12)
2D:4D RH		0.57			25.1	
		(0.82)			(34.76)	
2D:4D Avg			0.92			86.8*
			(0.89)			(33.83)
2D:4D LH sqr				-39.6**		
				(11.54)		
2D:4D RH sqr					-12.4	
					(17.66)	
2D:4D Avg sqr						-44.1*
						(17.38)
Constant	-0.82	-0.50	-0.84	-38.0**	-12.5	-42.6*
	(0.76)	(0.80)	(0.86)	(10.79)	(17.08)	(16.44)
N	330	330	330	330	330	330
F	16.0	15.1	15.7	15.9	10.7	14.2
p	0.00000023	0.00000053	0.00000031	1.1e-09	0.00000097	0.000000011

Robust standard errors in parentheses

* $p < 0.05$, ** $p < 0.005$

Table 9: Willingness to compete results, controlling for piece-rate task performance

	(1)	(2)	(3)	(4)	(5)	(6)
	Comp.	Comp.	Comp.	Comp.	Comp.	Comp.
2D:4D LH	1.24			60.3^{*}		
	(0.80)			(22.51)		
Piece-rate performance	0.024^{**}	0.024^{**}	0.024^{**}	0.022^{**}	0.024^{**}	0.023^{**}
	(0.01)	(0.01)	(0.01)	(0.01)	(0.01)	(0.01)
2D:4D RH		0.90			35.7	
		(0.86)			(36.58)	
2D:4D Avg			1.32			78.6^{*}
			(0.91)			(33.99)
2D:4D LH sqr				-30.5^{*}		
				(11.67)		
2D:4D RH sqr					-17.7	
					(18.58)	
2D:4D Avg sqr						-39.7^{*}
						(17.47)
Constant	-1.02	-0.70	-1.11	-29.6^{*}	-17.8	-38.7^{*}
	(0.78)	(0.85)	(0.89)	(10.84)	(18.00)	(16.52)
N	330	330	330	330	330	330
F	6.99	6.84	7.15	8.47	4.75	6.59
p	0.0011	0.0012	0.00091	0.000020	0.0030	0.00025

Robust standard errors in parentheses

* $p < 0.05$, ** $p < 0.005$

A.2 Dictator game giving studies

In a study looking at several social preference games, Buser (2012) runs a dictator game experiment and measures a binary indicator for choosing the selfish allocation compared to the altruistic allocation. This study uses a self-reported measure of 2D:4D, which has been criticised due to measurement error (see, e.g. Brañas-Garza and Kovarik (2013)). When splitting the sample into three groups with a dummy for low 2D:4D and a dummy for high 2D:4D (the reference group is therefore ring finger equal to index finger), the two dummy variables are not jointly statistically significant, for either hand. However there is a positive correlation between selfishness and the right hand ring finger being longer than the index finger (low 2D:4D), relative to the rest of the sample. The study finds the same positive correlation when both left and right hand ring finger is longer, but an insignificant result for just the left hand. Thus this study only finds statistically significant results when comparing low 2D:4D subjects to the rest of the sample (composed of intermediate and high 2D:4D).

While still in the context of a dictator game, Millet and Dewitte (2009) use a modified experimental strategy by priming mood (neutral or aggressive) in subjects and then measuring hypothetical dictator game giving, in a mixed gender sample. In the variation with a music video prime, they find a statistically significant negative association between right-hand 2D:4D and dictator game giving under the neutral condition and no relation under the aggressive condition (sample sizes for each of the conditions were unreported). In the second variation, using a language task with aggressive or neutral words, the result reverses with a positive correlation under the aggression condition and no result under the neutral condition.

Brañas-Garza et al. (2013) introduce the use of squared 2D:4D measures in this context and find conflicting results. Dictator game giving is statistically significantly positively associated with male, female and combined gender samples of right-hand 2D:4D, and in left hand 2D:4D in males and combined genders only. It is negatively correlated with male and both genders left and right hand squared 2D:4D and female right hand squared 2D:4D. They run the game again one year later with the same subjects but slightly smaller sample, and find fewer statistically significant results, as all effects have disappeared for the male only sample, and the left hand and left hand squared effect is insignificant for the combined gender sample. Right hand 2D:4D becomes statistically significant for the combined gender sample, showing a positive relationship with dictator game giving (although gender is not reported as a control in this regression).

Galizzi and Nieboer (2015) look at the digit ratio and dictator game giving in the context of different ethnic groups. They do not find a statistically significant correlation between dictator game giving and digit ratio in the full sample. Splitting the sample by ethnicity (Caucasian, Chinese and South Asian) and controlling for gender, they run OLS regressions on left and right hand 2D:4D separately, and then add the respective squared values of each in further regressions, resulting in 16 regression specifications. Of these, only the Caucasian subsample finds a statistically significant relationship, with a positive coefficient for right hand 2D:4D and a negative coefficient for right hand 2D:4D squared (the regression with right hand 2D:4D alone is not statistically significant).

Brañas-Garza et al. (2018a) use a large sample of 560 Caucasian subjects and measure dictator game giving (in addition to two other economic games). They find no statistically significant effect of 2D:4D on dictator game giving, when looking at both hands separately and controlling for gender in a combined gender sample (330 women, 230 men). They also find no statistically significant effect in

regressions with 2D:4D and 2D:4D squared, across left and right hands.

A.3 Risk taking studies

In an early paper investigating risk preferences and 2D:4D, Dreber and Hoffman (2007) measure risk-taking using a one-off risky investment decision. They report correlations between 2D:4D and risk for a Swedish sample, finding left hand 2D:4D is negatively correlated with risk taking after controlling for gender and ethnicity, but find no relationship for the right hand. However in a second American sample, they find no evidence for either hand. Also using a one-off risky investment decision, Apicella et al. (2008) find no statistically significant correlation between either left or right hand 2D:4D and risk preferences in a smaller, all male, sample. Subsequent literature has explored a variety of risk taking measures, as well as heterogeneous effects of ethnicity or gender using subsamples, and different specifications of the explanatory variables and controls.

Roughly half of the studies report all insignificant results, across a variety of 2D:4D specifications (left hand, right hand or average of both hands) and with some using subsamples (split by gender or ethnicity). Sapienza et al. (2009) find no statistically significant relationship between risk preferences and average 2D:4D in a mixed gender sample of 181. Running separate regressions for men and women, or for right and left-hand, also leads to insignificant results. Aycinena et al. (2014) look at an ethnically homogeneous sample from Guatamala and find no statistically significant association for either hand, and neither using specifications with squared 2D:4D. Splitting into subsamples by gender also yields insignificant results. Schipper (2014) measures only right hand 2D:4D, and risk preferences are measured in both the gain and loss domains (to control for framing effects) but there are no statistically significant results for men or women, even when using ethnically homogeneous subsamples (although these sample sizes are small). Similarly, Neyse et al. (2019) find no statistically significant results for left or right hand 2D:4D in either the gain domain, loss domain or mixed domain, across mixed-gender samples from Germany and Vietnam. Chicaiza-Becerra and Garcia-Molina (2017) use a sample from Colombia and follow the approach of Garbarino et al. (2011), but find no statistically significant correlations for either right or left hand 2D:4D and risk taking for both the full sample and a more ethnically homogeneous subsample. Lima de Miranda et al. (2018) find no statistically significant results for either left or right hand 2D:4D, as well as left and right hand 2D:4D squared. Finally, Alonso et al. (2018) assemble data from five experimental projects and the 2D:4D measure they use is a dummy variable which takes the value 1 if the subject has a 2D:4D measure above the gender-specific median value, and 0 otherwise. They look at the relationship between high 2D:4D and risky choices only for those subjects who made consistent decisions in the risk preference task, and find no statistically significant relationship for either right or left hand 2D:4D.

Other studies test multiple hypotheses and report mixed results, showing some statistically significant relationships with the sign in the hypothesised direction along with some insignificant findings. Sytsma (2014) uses the same methodology to measure risk preferences as in Garbarino et al. (2011), using a sample from Bangladesh. Two lotteries are employed: one with gains framing, the other with loss framing. Then as an additional measure, the average of the individual's choices over the 2 lotteries is used, and the digit ratio is tested for the left hand, right hand and average of both hands. The author finds significance in the same direction (negative association between 2D:4D and risk taking) for 6 out of the 27 specifications tested. In a study of 211 Israeli students, Barel (2017) looks at risk-taking moderated by optimism using self-reported risk measures, and finds a negative correlation between right hand 2D:4D and general risk taking, but no association for the left hand or for financial risk taking

(2D:4D is standardised within gender). Also using self-reporting, Stenstrom et al. (2011) use survey based measures of risk taking and measure right hand 2D:4D. They find a statistically significant negative correlation between financial risk taking and 2D:4D, and overall risk taking and 2D:4D, for the male subsample only.[15] They also look at a Caucasian subsample and find significance only for Caucasian males between overall risk taking and right hand 2D:4D. All results for females are insignificant.

Some studies limit to only one specification (such as one measure of 2D:4D and one risk taking measure using the full sample, although controls may differ) relating to 2D:4D and risk taking, although results are mixed. Garbarino et al. (2011) use normalised mean 2D:4D in a mixed-gender sample, controlling for both gain or loss framing and for other risk measures, and report one specification, finding a statistically significant negative relationship between 2D:4D and a risky choice task.[16] In a field experiment, Coates and Page (2009) look at a small sample of male traders. Using the standard deviation of their profit and loss over a 20 month period as the risk measure, they find that risk taken correlates negatively with right hand 2D:4D. In contrast, Drichoutis and Nayga (2015) use right hand and right hand squared 2D:4D and run a single regression specification. Their result finds no statistically significant relationship with risk taking, in a mixed-gender sample from Greece.

Whilst the hypothesised relationship between risk taking and 2D:4D is negative, one study finds a positive correlation. Brañas-Garza and Rustichini (2011) use two lottery choice tasks to create two measures of risk aversion for 188 caucasian subjects. Risk taking is positively correlated with 2D:4D for the female subsample of 116 participants, but insignificant for the male subsample. Their combined risk aversion (CRA) measure is insignificant for both genders, in a regression of CRA on right hand 2D:4D and a constant. In a simple correlation, CRA is positively correlated with 2D:4D for males only, not for females or the combined sample.

Two recent studies have the largest sample sizes across studies relating 2D:4D to risk preferences. Bönte et al. (2016) use a survey questionnaire on willingness to take risks in general, and in investment, instead of an experimentally elicited risk preference. They find that 2D:4D is negatively related to general risk taking but not to investment risk taking, for the right hand.[17] They find left hand 2D:4D to be uncorrelated with risk so do not report the full regression results for the left hand. In a larger study, Brañas-Garza et al. (2018b) use both self-reported risk attitude and lottery choices to measure risk preferences. In contrast to the finding in Bönte et al. (2016), Brañas-Garza et al. (2018b) do not find a statistically significant relationship between self-reported general risk and right hand 2D:4D (left hand is also insignificant). They do find a statistically significant negative relationship between both left and right hand 2D:4D and risk taking measured by the lottery choice task.

A.4 Willingness to compete studies

Two previous papers investigate the relationship between 2D:4D and the willingness to compete. Apicella et al. (2011) use self-selection into a competitive tournament or a piece rate scheme for payment in a maze solving task to measure willingness to compete. In an all male sample (83 left hand, 86 right hand), they find no association between left or right hand 2D:4D and the binary willingness

[15] The authors also look at recreational, social, ethical and health risk. They find significance only between recreational and social risk, and 2D:4D, for the Caucasian male subsample only.

[16] In addition, they explore non-linear effects using quartiles of the 2D:4D ratio.

[17] They also look at career risk and find no statistically significant relationship with right hand 2D:4D.

to compete measure. Using a similar task, and with larger sample sizes, Bönte et al. (2017), find no statistically significant results for either left or right hand 2D:4D, in two independent mixed-gender samples. They also use a self-reported competitiveness measure in addition to the experimentally elicited measure, and find a statistically significant negative relationship between right hand 2D:4D and self-reported competitiveness in both samples, but not for the left hand.

A.5 Economic Experiment Instructions

Instructions

In this computerized survey you will be faced with different decision making situations about real money. You will receive the money within 2 months.
Note that if you choose to send money to an organization, the money will also be sent there.

-----------New page-----------

DECISION MAKING SITUATION 1

Below you will be asked to make 5 different decisions of how to divide SEK 100 between yourself and a certain charity. One of these decisions will be randomly chosen to be paid out: the amount you choose to keep of the SEK 100 in this decision will be paid out to you, and the amount you allocate to the charity in this decision will be paid out to the charity at the end of the experiment.

First we want you to answer 3 control questions to make sure you understood how to answer the questions. Please click on the answer you think is correct. If you haven't answered the question correctly after two trials please raise your hand and you will receive help.

-----------New page-----------

CONTROL QUESTIONS

CONTROL QUESTION 1. If you choose to give SEK 0 of the SEK 100 to the charity, and this decision is chosen to be paid out, you get ...

□ … SEK 0 and the charity gets SEK 50.
□ … SEK 50 and the charity gets SEK 50.
□ … SEK 50 and the charity gets SEK 0.
□ … SEK 100 and the charity gets SEK 0.
□ … SEK 0 and the charity gets SEK 0.

CONTROL QUESTION 2. If you choose to give SEK 50 of the SEK 100 to the charity, and this decision is chosen to be paid out, you get …

□ … SEK 0 and the charity gets SEK 50.

□ … SEK 50 and the charity gets SEK 50.

□ … SEK 50 and the charity gets SEK 0.

□ … SEK 100 and the charity gets SEK 100.

□ … SEK 0 and the charity gets SEK 0.

CONTROL QUESTION 3. If you choose to give SEK 100 of the SEK 100 to the charity, and this decision is chosen to be paid out, you get …

□ … SEK 0 and the charity gets SEK 100.

□ … SEK 100 and the charity gets SEK 0.

□ … SEK 50 and the charity gets SEK 0.

□ … SEK 100 and the charity gets SEK 100.

□ … SEK 0 and the charity gets SEK 0.

-----------New page-----------

YOU WILL NOW MAKE YOUR DECISIONS

-----------New page-----------

[ONE DECISION PER PAGE IN THE EXPERIMENT]

1. You here decide how to **divide SEK 100** between yourself and **Stadsmissionen**. The decision is completely up to you. The money that you keep out of the SEK 100 will be paid to you and the money that you give to Stadsmisionnen will be sent there at the end of the experiment.

State the division of the SEK 100 below. The proposed division must sum up to SEK 100.

SEK to me _________

SEK to Stadsmissionen _________

2. You here decide how to **divide SEK 100** between yourself and **Röda Korset**. The decision is completely up to you. The money that you keep out of the SEK 100 will be paid to you and the money that you give to Röda Korset will be sent there at the end of the experiment.

State the division of the SEK 100 below. The proposed division must sum up to SEK 100.

SEK to me _________

SEK to Röda Korset _________

3. You here decide how to **divide SEK 100** between yourself and **Rädda Barnen**. The decision is completely up to you. The money that you keep out of the SEK 100 will be paid to you and the money that you give to Rädda Barnen will be sent there at the end of the experiment.

State the division of the SEK 100 below. The proposed division must sum up to SEK 100.

SEK to me _________

SEK to Rädda Barnen _________

4. You here decide how to **divide SEK 100** between yourself and **Radiohjälpen**. The decision is completely up to you. The money that you keep out of the SEK 100 will be paid to you and the money that you give to Radiohjälpen will be sent there at the end of the experiment.

State the division of the SEK 100 below. The proposed division must sum up to SEK 100.

SEK to me _________

SEK to Radiohjälpen _________

5. You here decide how to **divide SEK 100** between yourself and **Cancerfonden**. The decision is completely up to you. The money that you keep out of the SEK 100 will be paid to you and the money that you give to Cancerfonden will be sent there at the end of the experiment.

State the division of the SEK 100 below. The proposed division must sum up to SEK 100.

SEK to me _________

SEK to Cancerfonden _________

-----------New page-----------

DECISION MAKING SITUATION 2

In this part you will be faced with 18 choices. For each choice you will choose between getting certain amount of money **with certainty** and to **flip a coin** to win a **specific amount of money**. The amount that you can win in the coin flip varies between SEK 200 and SEK 400 in the different questions.

When you have answered all 18 questions, one of the questions will be chosen to be paid out. If you in the chosen question chose money with certainty, you will get this money. If you chose the coin flip, it will be decided (with a coin flip) whether you won or not (head implies gain and tails implies loss).

First we want you to answer 2 control questions to make sure you understood the experiment.

Please click on the answer you think is correct. If you haven’t answered the question correctly after two trials please raise your hand and you will receive help.

-----------New page-----------

CONTROL QUESTIONS

CONTROL QUESTION 1. If you choose between SEK 80 with certainty and to flip a coin where you can win SEK 200 and you choose the coin flip, and this decision is chosen to be paid out with real money, then you get…

□ … SEK 80
□ … SEK 80 if the coin comes up heads
□ … SEK 200 if the coin comes up heads
□ … SEK 200 if the coin comes up tails
□ … SEK 200 kronor no matter if the coin comes up heads or tails

CONTROL QUESTION 2. If you choose between SEK 80 with certainty and to flip a coin where you can win SEK 200 and you choose SEK 80 with certainty, and this decision is chosen to be paid out with real money, then you get…

□ … SEK 80
□ … SEK 80 if the coin comes up heads
□ … SEK 200 if the coin comes up heads
□ … SEK 200 if the coin comes up tails
□ … SEK 200 kronor no matter if the coin comes up heads or tails

-----------New page-----------

1. Which option do you choose:
______SEK 160
______to flip a coin to win SEK 400 (heads) or SEK 0 (tails)

-----------New page-----------

2. Which option do you choose:
______SEK 120
______ to flip a coin to win SEK 200 (heads) or SEK 0 (tails)

-----------New page-----------

3. Which option do you choose:

______SEK 90

______ to flip a coin to win SEK 300 (heads) or SEK 0 (tails)

-----------New page-----------

4. Which option do you choose:

______SEK 180

______ to flip a coin to win SEK 300 (heads) or SEK 0 (tails)

-----------New page-----------

5. Which option do you choose:

______SEK 100

______ to flip a coin to win SEK 200 (heads) or SEK 0 (tails)

-----------New page-----------

6. Which option do you choose:

______SEK 120

______ to flip a coin to win SEK 400 (heads) or SEK 0 (tails)

-----------New page-----------

7. Which option do you choose:

______SEK 120

______ to flip a coin to win SEK 300 (heads) or SEK 0 (tails)

-----------New page-----------

8. Which option do you choose:

______SEK 240

______ to flip a coin to win SEK 400 (heads) or SEK 0 (tails)

-----------New page-----------

9. Which option do you choose:

______SEK 200

______ to flip a coin to win SEK 400 (heads) or SEK 0 (tails)

-----------New page-----------

10. Which option do you choose:

______SEK 80

______ to flip a coin to win SEK 200 (heads) or SEK 0 (tails)

-----------New page-----------

11. Which option do you choose:

______SEK 150

______ to flip a coin to win SEK 300 (heads) or SEK 0 (tails)

-----------New page-----------

12. Which option do you choose:

______SEK 60

______ to flip a coin to win SEK 200 (heads) or SEK 0 (tails)

-----------New page-----------

13. Which option do you choose:

______SEK 60

______ to flip a coin to win SEK 300 (heads) or SEK 0 (tails)

-----------New page-----------

14. Which option do you choose:

______SEK 40

______ to flip a coin to win SEK 200 (heads) or SEK 0 (tails)

-----------New page-----------

15. Which option do you choose:

______SEK 280

______ to flip a coin to win SEK 400 (heads) or SEK 0 (tails)

-----------New page-----------

16. Which option do you choose:

______SEK 140

______ to flip a coin to win SEK 200 (heads) or SEK 0 (tails)

-----------New page-----------

17. Which option do you choose:

______SEK 210

______ to flip a coin to win SEK 300 (heads) or SEK 0 (tails)

-----------New page-----------

18. Which option do you choose:

______SEK 80

______ to flip a coin to win SEK 400 (heads) or SEK 0 (tails)

-----------New page-----------

DECISION MAKING SITUATION 2

This part consists of three subparts. In all three subparts you will solve simple arithmetic problems. You can see an example of the exactly same type below. In each part you are to solve as many problems as you can during 3 minutes.

1	**63**	+	**19**	+	**12**	+	**44**	=	……

Subpart 1: You get 3 minutes to solve as many problems as possible. For each problem solved correctly you earn SEK 5.

-----------New page-----------

Subpart 1:

1	**31**	+	**12**	+	**21**	+	**21**	=	……
2	**27**	+	**30**	+	**24**	+	**6**	=	……
3	**18**	+	**27**	+	**10**	+	**31**	=	……
4	**10**	+	**28**	+	**17**	+	**10**	=	……
5	**25**	+	**4**	+	**26**	+	**20**	=	……
Etc									

-----------New page-----------

Subpart 2: You will now compete against another person who has previously participated in the experiment. As in part 1 you will get 3 minutes to solve as many problems as possible. You will earn SEK 10 per problem if you solve more problems than the person you compete against. Otherwise you get nothing. If there is a tie, you get SEK 5 per solved problem.

-----------New page-----------

Subpart 2:

1	**31**	+	**12**	+	**21**	+	**21**	=	……
etc									……

-----------New page-----------

<u>Subpart 3.</u> You get 3 minutes to solve problems here too. Now you get to decide which of the 2 following ways you want to get paid.

1) Payment as in part 1: **SEK 5** for each problem you solve correctly.
2) Payment as in part 2: **SEK 10** for each correctly solved problem if you solve more problems than the person you compete against solved in subpart 2. If you solve fewer you earn SEK 0. If there is a tie, you get SEK 5 per solved problem.

Please click on the payment method that you prefer.

1) **2)**

-----------New page-----------

<u>Subpart 3:</u>

1	**31**	+	**12**	+	**21**	+	**21**	=	……
etc									……
