# Supplementary material for: The digit ratio (2D:4D) and economic preferences: no robust associations in a sample of 330 women
Source: J Econ Sci Assoc. 2019 Oct 11;5(2):149–69. doi: 10.1007/s40881-019-00076-y (PMC6917633; doi:10.1007/s40881-019-00076-y)
Supplement: Supplementary file 2 — Supplementary material 2 (pdf 313 KB) [file 40881_2019_76_MOESM2_ESM.pdf]

B Online Appendix

B.1 Non-Economic Experiment Instructions

20120130 only health

Start of Block: Drug no

☐

Q153 Enter your patient number:

____

End of Block: Drug no

Start of Block: Log of sexual activity

Q183 LOGO **ABOUT SEXUAL ACTIVITY INSTRUCTIONS FOR COMPLETING THE LOGO ON SEXUAL ACTIVITY** This logbook on sexual activity contains questions about your physical health and sexual activities over the past seven days. In this survey, sexual activity can mean any of the following: Masturbation (with or without your partner), intercourse, giving or receiving oral sex, and other sexual activities. This information will be used to study treatments for women who have lost any sexual function . By completing the survey you will help with that work. A big thank you for your time and effort in filling out this activity blog!

Q184 **YOUR PHYSICAL HEALTH** First, we want to ask you about your physical health in general and then, more specifically, about your physical health **over the past seven days.** ***(Click only one option on each line)***

	Bad (1)	Fairly good (2)	Good (3)	Very good (4)	Excellent (5)
Estimate your general physical health: (1)	o	o	o	o	o

2 (2)	O	O	O	O	O

Q262 ***(Click only one option on each line)***

	Bad (1)	Fairly good (2)	Good (3)	Very good (4)	Excellent (5)
Estimate your physical health over the past few days: (1)	O	O	O	O	O

Q186 ***Over the past seven days, was your physical health or desire for sexual activity negatively affected by your physical health?***

- O Yes (1)
- O No (2)

Q187 **PARTNER AVAILABILITY Over the past seven days, how many days was your partner available for sexual activity?**

____

Page Break

Q188 LOGO **ABOUT SEXUAL ACTIVITY (Continued) LOGO ABOUT SEXUAL ACTIVITY The** following questions are about your sexual activities over the past seven days. **Section 1** - Refers to sexual activity where intercourse ***was included*** as part of the activity. **Section 2** - Refers to sexual activity where sexual intercourse is ***not*** part of the activity. This includes activities such as giving or receiving oral sex, masturbation with or without your partner, or any other activity where intercourse did not occur. Please answer each question below by entering numbers only in the space provided. Type 0 if the activity did not take place. Do not leave any response space blank.

Q189 **Section 1: Sexual Activities Including Sexual Intercourse** 1. **In the past seven days,** how many times have you participated in a sexual activity involving sexual intercourse?

____

Q190 2. **Over the past seven days,** how many orgasms did you experience during sexual activities involving intercourse?

____

Q191 3. **Over the past seven days,** how many times were these sexual activities involving intercourse satisfactory to you?

____

Q192 **Section 2: Sexual Activities in which Sexuality was Not Included The following questions relate to sexual activities that took place during the last seven days where sexual intercourse was not included . These activities may include giving and receiving oral sex, masturbation (with or without your partner) or other sexual activity.** 4. **Over the past seven days,** how many times have you engaged in sexual activities that *did not include* sexual intercourse ?

____

Q193 5. **Over the past seven days,** how many orgasms did you experience during sexual activities where intercourse was ***not*** included?

____

Q194 6. **Over the past seven days,** how many times were these sexual activities where intercourse was ***not*** satisfactory ***to you*** ?

____

Q195 By signing this form with my initials and dated it, I confirm that I have completed the questionnaire.

initials:

____

Q196 Date: (Example: 14-NOV-2000)

____

End of Block: Log of sexual Akti efficiency

Start or Block: PFSF

Q197 PFSF **QUESTION PAPER INSTRUCTIONS FOR THE QUESTION PAPER This questionnaire asks questions about your sexual feelings and activities *over the past 30 days* . In this questionnaire, sexual activities can include all or any of the following: sexual thoughts and fantasies, passionate kisses, masturbation, sexual foreplay and touch, giving or receiving oral sex, and intercourse. This information will be used to study treatment of women who have lost some sexual function. By completing this questionnaire, you will assist in this work. Please take the time to read and answer each question carefully. Pay attention to the rating scale. Some questions may be perceived as similar, but each one is different from the other. *Here is an EXAMPLE of how most of the questions in this form will look like:***

	Always (1)	Very often	Often (3)	Sometimes	Rarely (5)	Never (6)

		(2)		(4)		
1. I am happy with my sex life. (1)	O	O	O	O	O	O

Q209 THANK **YOU SO MUCH FOR TAKING YOUR TIME AND HAVING YOUR DIFFICULTY TO FILL IN THIS QUESTION FORM!**

Page Break

Q198 PFSF **QUESTION PAPER (Continued) YOUR SEXUALITY IN GENERAL First, we would like to ask a question about your sexuality in general. If you think about the last 30 days , how would you rate** your overall satisfaction with your sexuality ***(Click on one option only)***

- Bad (1)
- Something (2)
- Good (3)
- Very good (4)
- Excellent (5)

Page Break

Q199 PFSF **QUESTION PAPER (Continued) SEXLUST: The** following questions are about feelings related to sexual desire and interest in sexual activities. The feeling can be either mental or physical and can arise during periods without sexual activities. Read each statement carefully and click on the option that best matches how you have felt over the past 30 days . Remember that sex refers to all sexual activities, not just intercourse. **How often did the following statements fit you in the last 30 days :** *(Click an option on each line)*

	Always (1)	Very often (2)	Often (3)	Sometimes (4)	Rarely (5)	Never (6)
1. I wanted to see x (1)	O	O	O	O	O	O
2. I had great sexual desire (2)	O	O	O	O	O	O
3. I really wanted sex (3)	O	O	O	O	O	O
4. I had sexual desire (4)	O	O	O	O	O	O
5. I lacked sexual desire (5)	O	O	O	O	O	O
6. I had strong sexual feelings (6)	O	O	O	O	O	O
7. I was uninterested in sex (7)	O	O	O	O	O	O
8. I got hot in my body just thinking about sex (8)	O	O	O	O	O	O
9. I was really sexually excited (9)	O	O	O	O	O	O

Q200 **PFSF QUESTION PAPER (Continued) SEXUAL EXCITATION: The** following questions are about how easy it is to get sexually aroused, either physically or emotionally. Read each statement carefully and click on the option that best matches how you have felt over the past 30 days . Keep in mind that sex refers to all sexual activities, not just sexual intercourse. **How often did the following statements fit you in the last 30 days :** *(Click an option on each line)*

	Always (1)	Very often (2)	Often (3)	Sometimes (4)	Rarely (5)	Never (6)
1. It is very difficult for me to get excited (1)	O	O	O	O	O	O
2. I had to really concentrate on getting sexually excited (2)	O	O	O	O	O	O
3. It took ages to get excited (3)	O	O	O	O	O	O

Q201 **ORGASM: The** following questions are about the ability to have an orgasm. Read each statement carefully and click on the option that best matches how you have felt over the past 30 days . **How often have the following statements been applied to you in the last 30 days :** *(Click an option on each row)*

	Always (1)	Very often (2)	Often (3)	Sometimes (4)	Rarely (5)	Never (6)
1. I had easy orgasm (1)	O	O	O	O	O	O
2. It took a lot of work to get	O	O	O	O	O	O

orgasm (2)						
3. It was difficult to get orgasm (3)	O	O	O	O	O	O
4. It was impossible to have orgasm (4)	O	O	O	O	O	O

Page Break

Q202 PFSF **QUESTION PAPER (Continued) SEXUAL ENJOYMENT: The** following questions are about feelings of sexual pleasure. Read each statement carefully and click on the option that best matches how you have felt over the past 30 days . Remember that sex refers to all sexual activities, not just sexual intercourse. **How often did the following statements fit you in the last 30 days :** *(Click an option on each line)*

	Always (1)	Very often (2)	Often (3)	Sometimes (4)	Rarely (5)	Never (6)
1. I enjoyed sex a lot (1)	O	O	O	O	O	O
2. Sex felt good (2)	O	O	O	O	O	O
3. I felt warm throughout my body at six (3)	O	O	O	O	O	O
4. Sex was wonderful (4)	O	O	O	O	O	O
5. It pierced the whole body at six (5)	O	O	O	O	O	O
6. Sex was exciting (6)	O	O	O	O	O	O
7. Sex was satisfactory (7)	O	O	O	O	O	O

Q203 **SEXUAL CONCERNS: The** following questions are about concerns you may have regarding your sexuality. Read each statement carefully and click on the option that best matches how you have felt over the past 30 days . **How often did the following statements fit in on you in the last 30 days :** *(Click an option on each row)*

	Always (1)	Very often (2)	Often (3)	Sometimes (4)	Rarely (5)	Never (6)
1. I was frustrated with my sex life (1)	O	O	O	O	O	O
2. I was sexually assaulted (2)	O	O	O	O	O	O
3. I felt worried about sex (3)	O	O	O	O	O	O

Page Break

Q204 PFSF **QUESTION PAPER (Continued) SEXUAL RECOVERY: The** following questions are about the efforts you may have made to initiate or avoid sex, or feelings you may have had for wanting to initiate or avoid sex. Read each statement carefully and click on the option that best matches how you have felt over the past 30 days . **How often did the following statements fit you in the last 30 days :** *(Click an option on each line)*

	Always (1)	Very often (2)	Often (3)	Sometimes (4)	Rarely (5)	Never (6)
1. I enjoyed the initiative to six (1)	O	O	O	O	O	O
2. I welcomed the chance to have sex (2)	O	O	O	O	O	O
3. I found excuses for not having sex (3)	O	O	O	O	O	O
4. I avoided doing things that would get my partner sexually excited (4)	O	O	O	O	O	O
5. I Avoid Having Sex (5)	O	O	O	O	O	O
6. I trembled to have sex (6)	O	O	O	O	O	O
7. Sex felt like an obligation (7)	O	O	O	O	O	O

Q205 SEXUAL **SELF PICTURE: The** following questions are about how you felt about your own sexuality. Read each statement carefully and click on the option that best matches how you have felt over the past 30 days . **How often have you been in the last 30 days :** *(Click on an option on each row)*

	Always (1)	Very often (2)	Often (3)	Sometimes (4)	Rarely (5)	Never (6)
1. Satisfied with yourself purely sexual (1)	O	O	O	O	O	O
2. Sexually desirable (2)	O	O	O	O	O	O
3. As a sensual woman (3)	O	O	O	O	O	O
4. Sexually dissatisfied with yourself (4)	O	O	O	O	O	O

Q206 By writing my initials and dates on this form, I confirm that I have completed PFSF.

initials:

____

Q207 Date: (Example: 14-NOV-2000)

____

End of Block: PFSF

Start or Block: PDS

Q208 **PDS SURVEY INSTRUCTIONS FOR COMPLETING THE SURVEY This survey contains questions about how you felt your interest in sex was in the last 30 days. Take the time to carefully read and answer each question. Look carefully at the estimation scale. Some of the questions may be similar to others, but each question is unique. Complete the questionnaire in a place you feel comfortable, quiet and secluded. You should fill in the entire questionnaire without interruption. Below is an EXAMPLE of what**

most questions will look like:

	Always (1)	Very often (2)	Often (3)	Sometimes (4)	Rarely (5)	Never (6)
1. I was not satisfied with my interest in sex (1)	O	O	O	O	O	O

Q210 **BIG THANKS FOR YOUR TIME AND WORKED TO ANSWER THIS SURVEY!**

Page Break

Q211 **PDS SURVEY YOUR FEELINGS ABOUT SEX:** These questions are about how you experienced your interest in sex. Carefully read each statement and click on the option that best matches how you felt over the past 30 days. Remember that sex refers to all sexual activities and not just sexual intercourse. **How often during the last 30 days did the following statements apply to you:** *(Click on an altarnative on each line)*

	Always (1)	Very often (2)	Often (3)	Sometimes (4)	Rarely (5)	Never (6)
1. I was concerned about my lack of interest in sex (1)	O	O	O	O	O	O
2. I was unhappy with my lack of interest in sex (2)	O	O	O	O	O	O
3. I was disappointed with my lack of interest in sex (3)	O	O	O	O	O	O
4. I was upset by my lack of interest in sex (4)	O	O	O	O	O	O
5. I felt frustrated at my lack of interest in sex (5)	O	O	O	O	O	O
6. I felt sorry for my lack of interest in sex (6)	O	O	O	O	O	O
7. I was worried about my	O	O	O	O	O	O

lack of interest in sex (7)

Q212 By signing this form with my initials and dated it, I confirm that I have completed the survey.

initials:

____

Q213 Date: (Example: 14-NOV-2000)

____

End of Block: PDS

Start of Block: PGWB

Q214 **PSYCHOLOGICAL GENERAL WELL-BEING INDEX The** following pages contain questions about how you are doing and how you have been doing it during the past month. Indicate by clicking which option best fits you and your situation. **1. How have you generally felt in the past month?** (Click on an option)

- 5 In an excellent mood (1)
- 4 In very good mood (2)
- 3 Mostly in a good mood (3)
- 2 The mood has varied (4)
- 1 Mostly in a bad mood (5)
- 0 In very bad mood (6)

Q215 **2. Have you been afflicted with illness, physical illness, pain or pain in the past month?** (Click on an option)

- 0 All the time (1)

- 1 Most of the time (2)
- 2 A lot of the time (3)
- 3 Part of the time (4)
- 4 A Little Time (5)
- 5 None of the time (6)

Q216 **3. Have you felt down in the past month?** (Click on an option)

- 0 Yes, to the extent that I felt that life is not worth living (1)
- 1 Yes, to the extent that I did not care about anything (2)
- 2 Yes, very depressed almost every day (3)
- 3 Yes, quite downplayed on several occasions (4)
- 4 Yes, a little jumbled now and then (5)
- 5 No, not at all depressed (6)

Page Break

Q217 **PGWB QU ESTIONNAIRE (Continued) 4. Have you had good control over your behavior, your thoughts and feelings during the past month?** (Click on an option)

- 5 Yes, definitely (1)
- 4 Yes, mostly (2)
- 3 In general (3)
- 2 Not very good (4)
- 1 No, and it is quite disturbing (5)
- 0 No, and it is very disturbing (6)

Q218 **5. Have you been feeling nervous or worried in the past month?** (Click on an option)

- 0 Extremely high - to the extent that I could not handle everyday chores (1)
- 1 Very much (2)
- 2 A great deal (3)
- 3 Some - enough to worry me (4)
- 4 Little grand (5)
- 5 Not at all (6)

Q219 **6. Have you felt energetic, vibrant and vital in the past month?** (Click on an option)

- 5 Full of energy - giant spikes (1)
- 4 Most energetic (2)
- 3 My energy and vitality have varied (3)
- 2 Not so bad or energetic (4)

- 1 Mostly dull and largely out of energy (5)
- 0 No energy and vitality at all - I have felt completely leached and ready (6)

Page Break

Q220 **PGWB QUESTIONNAIRE (Continued) 7. I have felt sad and discouraged over the past month.** (Click on an option)

- 5 None of the time (1)
- 4 A Little Time (2)
- 3 Part of the time (3)
- 2 A lot of the time (4)
- 1 Most of the time (5)
- 0 All the time (6)

Q221 **8. Have you felt tense in the past month?** (Click on an option)

- 0 Extremely tense all the time (1)
- 1 Mostly very tense (2)
- 2 Quite tense on several occasions (3)
- 3 A little tense now and then (4)
- 4 Not very tense (5)
- 5 Not at all tense (6)

Q222 **9. Have you felt happy, satisfied and satisfied with life during the past month?** (Click on an option)

- 5 Extremely Happy - I Could Not Be More Satisfied and Satisfied (1)
- 4 Mostly very happy (2)
- 3 generally happy and satisfied (3)
- 2 Sometimes happy - sometimes unhappy (4)

- 1 Generally unhappy and unsatisfied (5)
- 0 Always or mostly very unsatisfied and unhappy (6)

Pa ge Break

Q223 **PGWB QUESTIONNAIRE (Continued) 10. Have you felt so healthy that you have been able to do what you want or need to do in the last month?** (Click on an option)

- 5 Yes, definitely (1)
- 4 Yes, mostly (2)
- 3 My health has severely restricted me (3)
- 2 I've only been able to take care of myself (4)
- 1 I've Needed A Lot of Help to Succeed (5)
- 0 I needed help with pretty much everything (6)

Q224 **11. Have you felt so sad, discouraged, or without hope that you have wondered if anything at all has been meaningful in the past month?** (Click on an option)

- 0 Extremely much, to the extent that I was ready to give up (1)
- 1 Very much (2)
- 2 A great deal (3)
- 3 Some - enough to worry me (4)
- 4 Little grand (5)
- 5 Not at all (6)

Q225 **12. I have felt fresh and rested when I woke up during the past month.** (Click on an option)

- 0 None of the time (1)
- 1 A Little Time (2)
- 2 Part of the time (3)

- 3 A lot of the time (4)
- 4 Most of the time (5)
- 5 All the time (6)

Page Break

Q226 **PGWB QUESTIONNAIRE (Continued) 13. Have you been concerned or worried about your health in the past month?** (Click on an option)

- 0 Extremely High (1)
- 1 Very much (2)
- 2 A great deal (3)
- 3 Part (4)
- 4 Little grand (5)
- 5 Not at all (6)

Q227 **14. Did you feel like you were losing your mind or losing control of your feelings, thoughts, and actions during the past month?** (Click on an option)

- 5 Not at all (1)
- 4 Only a little grand (2)
- 3 Some, but not so much that it worries or worries me (3)
- 2 Some and it has worried me a bit (4)
- 1 A lot and it worried me a lot (5)
- 0 Yes, very much and I'm very worried (6)

Q228 **15. My life has been filled with things that interest me over the past month.** (Click on an option)

- 0 None of the time (1)
- 1 A Little Time (2)
- 2 Part of the time (3)

- 3 A lot of the time (4)
- 4 Most of the time (5)
- 5 All the time (6)

Page Break

Q229 **PGWB QUESTIONNAIRE (Continued) 16. Have you felt active and energetic or dull and hungry during the past month?** (Click on an option)

- 5 All the time very active and energetic (1)
- 4 Mostly active and energetic - never really dull and hungry (2)
- 3 Quite active and energetic - rarely lethargic and hungry (3)
- 2 Quite lethargic and hungry - rarely active and energetic (4)
- 1 Mostly dull and hung - never really active and energetic (5)
- 0 Very lethargic and hungry all the time (6)

Q230 **17. Have you felt worried, upset or anxious in the past month?** (Click on an option)

- 0 Extremely high - to the extent that I felt sick from worry (1)
- 1 Very much (2)
- 2 A great deal (3)
- 3 Some - enough to worry me (4)
- 4 Little grand (5)
- 5 Not at all (6)

Q231 **18. I have felt in balance and confident in myself for the past month.** (Click on an option)

- 0 None of the time (1)
- 1 A Little Time (2)
- 2 Part of the time (3)
- 3 A lot of the time (4)

- 4 Most of the time (5)
- 5 All the time (6)

Page Break

Q232 **PGWB QUESTIONNAIRE (Continued) 19. Have you felt relaxed and calm or stressed, tense and screwed up during the past month?** (Click on an option)

- 5 Relaxed and calm all the time (1)
- 4 Mostly relaxed and calm (2)
- 3 Usually calm but occasionally quite tense (3)
- 2 Most often tense but occasionally relaxed (4)
- 1 Mostly stressed, strained and screwed up (5)
- 0 All the time stressed, tense and screwed up (6)

Q233 **20. I have felt happy and carefree in the last month.** (Click on an option)

- 0 None of the time (1)
- 1 A Little Time (2)
- 2 Part of the time (3)
- 3 A lot of the time (4)
- 4 Most of the time (5)
- 5 All the time (6)

Q234 **21. I have felt tired and exhausted in the last month.** (Click on an option)

- 5 None of the time (1)
- 4 A Little Time (2)
- 3 Part of the time (3)
- 2 A lot of the time (4)

- 1 Most of the time (5)
- 0 All the time (6)

Page Break

Q235 **PGWB QUESTIONNAIRE (Continued) 22. Have you felt stressed, pressured or jaded during the past month?** (Click on an option)

- 0 Yes, at the limit of what I have been able to do (1)
- 1 A lot of stress (2)
- 2 Some - more than usual (3)
- 3 Some - much like usual (4)
- 4 Little grand (5)
- 5 Not at all (6)

Q236 By signing this form with my initials and dated it, I confirm that I have completed the survey.

initials:

________ ____

Q237 Date: (Example: 14-NOV-2000)

____

End of Block: PGWB

Start of Block: BDI

Q239 Here are 21 groups of claims. Read each grid carefully and then click for the option (0, 1, 2 or 3) that belongs to the statement in each group, which *best* describes how you have felt over *the past week, including today* . If more than one statement fits equally well, you should call in all of them . *Be sure to read all the statements in a group before making your choice.*

Q240 1.

- 0 I feel neither sad nor depressed. (1)
- 1 I feel sad and depressed. (2)

- 2 I feel sad and depressed all the time, and can't break out of it. (3)
- 3 I'm so sorry and unhappy that I can't stand it. (4)

Q241 2.

- 0 I don't feel particularly discouraged for the future. (1)
- 1 I feel discouraged for the future. (2)
- 2 I feel I have nothing to look forward to. (3)
- 3 I feel that the future is hopeless and that things cannot be improved. (4)

Q242 3.

- 0 I do not feel unsuccessful. (1)
- 1 I feel I have failed more than most. (2)
- 2 When I look back on my life, I can only see a multitude of failures. (3)
- 3 I feel totally unsuccessful as a human being. (4)

Page Break

Q243 4.

- 0 I am as satisfied with things as before. (1)
- 1 I do not appreciate things in the same way as before. (2)
- 2 I'm not really happy with anything these days. (3)
- 3 I am bored and dissatisfied with everything. (4)

Q244 5.

- 0 I don't feel particularly guilt conscious. (1)
- 1 I often feel guilt-conscious. (2)
- 2 I feel mostly guilt conscious. (3)
- 3 I feel constantly guilt conscious. (4)

Q245 6.

- 0 I don't feel like I'm being punished. (1)
- 1 I have a feeling that I can be punished. (2)
- 2 I expect to be punished. (3)
- 3 I feel that I am being punished. (4)

Q246 7.

- 0 I do not feel disappointed in myself. (1)
- 1 I am disappointed in myself. (2)
- 2 I feel a strong dislike for myself. (3)

- 3 I hate myself. (4)

Q247 8.

- 0 I do not feel that I am inferior to anyone else. (1)
- 1 I criticize myself for my weaknesses or mistakes. (2)
- 2 I am constantly blaming myself for my mistakes and shortcomings. (3)
- 3 I blame myself for everything negative that happens. (4)

Q248 9.

- 0 I have no thoughts on killing me. (1)
- 1 I have thoughts of killing myself, but would never. (2)
- 2 I would like to take my life. (3)
- 3 I would take my life if I had the opportunity. (4)

Q249 10.

- 0 I cry no more than usual. (1)
- 1 I cry more now than before. (2)
- 2 I am still crying nowadays. (3)
- 3 I used to cry before, but now I can't, though I would. (4)

Page Break

Q250 11.

- 0 I am no more annoyed now than usual. (1)
- 1 I get annoyed or annoyed more easily now than before. (2)
- 2 I feel equally annoyed nowadays. (3)
- 3 Nowadays I am often annoyed by things that never annoyed me before. (4)

Q251 12.

- 0 I have not lost interest in other people. (1)
- 1 I'm less interested in people now than before. (2)
- 2 I have lost most of my interest in other people. (3)
- 3 I have lost all interest in other people. (4)

Q252 13.

- 0 I can make decisions just as well as before. (1)
- 1 I postpone decisions to a greater extent than before. (2)
- 2 I have more difficulty making decisions nowadays. (3)
- 3 I can't make decisions at all nowadays. (4)

Q253 14.

- 0 I don't think I look worse now than before. (1)
- 1 I worry because I look old or unattractive. (2)
- 2 I feel that my appearance has undergone such lasting changes that I am now

unattractive. (3)

- 3 I think I'm full. (4)

Q254 15.

- 0 I can work just as well as before. (1)
- 1 It takes extra effort to get started and do something. (2)
- 2 I have to push myself hard to do anything. (3)
- 3 I can't work at all. (4)

Q255 16.

- 0 I sleep as well as before. (1)
- 1 I sleep worse than before. (2)
- 2 I wake up 1-2 hours earlier than usual, and have trouble falling asleep. (3)
- 3 I wake up several hours earlier than usual and can't possibly fall asleep. (4)

Page Break

Q256 17.

- 0 I'm not getting tired now than usual. (1)
- 1 I'm getting tired more easily now than before. (2)
- 2 I get tired of the smallest. (3)
- 3 I'm too tired to do anything at all. (4)

Q257 18.

- 0 I have no worse appetite than usual t. (1)
- 1 I do not have as good appetite as usual. (2)
- 2 I have much worse appetite than usual. (3)
- 3 I have no appetite at all now. (4)

Q258 19.

- 0 I haven't exactly lost weight lately. (1)
- 1 I have lost more than 2 kg. (2)
- 2 I have lost more than 5 kg. (3)
- 3 I have lost more than 7 kg. (4)

Q259 I try to consciously lose weight by eating less.
Click for the correct answer!

- Yes (1)
- No (2)

Q260 20.

- 0 I'm not worried about my health now than before. (1)
- 1 I am concerned about physical problems, such as pain and pain, upset stomach or constipation. (2)
- 2 I am very concerned about my health and find it hard to think of anything else. (3)
- 3 I'm so worried about my health that I can't think of anything else. (4)

Q261 21.

- 0 I have not noticed any changes in my interest in sex. (1)
- 1 I'm less interested in sex now than before. (2)
- 2 I'm much less interested in sex nowadays. (3)
- 3 I've completely lost interest in sex. (4)

Page Break

Q 263 By signing this form with my initials and dated it, I confirm that I have completed the questionnaire.

initials:

____

Q264 Date: (Example: 14-NOV-2000)

____

End of Block: BDI

Start of Block: FACE PREFERENCES

Q87 DECISION SITUATION 7 In this section you will be confronted with 12 different pairs of faces. For each couple, we will ask you which face you find most attractive.

Page Break

Q100 **NOW YOU MUST MAKE YOUR DECISION**

Page Break

Q88 1. Which face do you find most attractive?

- ○ Image: Masc male6 0 ca (5)
- ○ Image: Masc male6 1 ca (6)

Page Break

Q89 2. Which face do you find most attractive?

- Image: Sym male3 1 ca (6)
- Image: Sym male3 0 ca (7)

Page Break

Q90 3. Which face do you find most attractive?

- Image: Sym male2 1 ca (5)
- Image: Sym male2 0 ca (6)

Page Break

Q91 4. Which face do you find most attractive?

- ○ Image: Sym male4 1 ca (5)
- ○ Image: Sym male4 0 ca (6)

Page Break

Q92 5. Which face do you find most attractive?

- Image: Masc male2 0 ca (5)
- Image: Masc male2 1 ca (6)

Page Break

Q93 6. Which face do you find most attractive?

- ○ Image: Sym male1 1 ca (5)
- ○ Image: Sym male1 0 ca (6)

Page Break

Q94 7. Which face do you find most attractive?

- Image: Masc male5 1 ca (7)
- Image: Masc male5 0 ca (8)

Page Break

Q95 8. Which face do you find most attractive?

- ○ Image: Masc male1 1 ca (5)
- ○ Image: Masc male1 0 ca (6)

Page Break

Q96 9. Which face do you find most attractive?

- Image: Sym male5 1 ca (5)
- Image: Sym male5 0 ca (6)

Page Break

Q97 10. Which face do you find most attractive?

- Image: Masc male3 0 ca (5)
- Image: harsh (7)

Page Break

Q98 11. Which face do you find most attractive?

- Image: Masc male4 1 ca (5)
- Image: Masc male4 0 ca (6)

Page Break

Q99 12. Which face do you find most attractive?

- ○ Image: Sym male6 0 ca (5)
- ○ Image: Sym male6 1 ca (6)

Page Break

End of Block: FACE PREFERENCES
